# Supplementary material for: Construction and practice of a novel pharmaceutical health literacy intervention model in psychiatric hospital
Source: PLoS One. 2024 Oct 23;19(10):e0311841. doi: 10.1371/journal.pone.0311841 (PMC11498655; doi:10.1371/journal.pone.0311841)
Supplement: Attachment — Submitted filename: Response to Reviewers.docx [file pone.0311841.s002.docx]

Dear editor/reviewers,

We apologize for the delayed response. We greatly appreciate your constructive feedback and professional advice. These suggestions have helped us enhance the academic rigor of our article. We have thoroughly revised the manuscript in response to the comments from the editors and reviewers.

In the **Introduction** section, we included a literature review, refined the explanation of the "chunk-check" method, and thoroughly reorganized the structure. In the **Methods** section, we improved the content regarding pharmacists' qualifications and training, introduced the use of WeChat public account and instant messaging software (WeChat), and provided a more detailed overview of the statistical analysis methods. In the **Results** section, we increased the patient pool for PHLI and revised unclear statements.

Finally, we apologize for the poor language quality and have conducted a thorough language revision. We have uploaded the marked manuscript, with the page numbers in parentheses corresponding to the simplified revision mode.

Additional Editor Comments:

Based on the referees' recommendations and comments, I recommend Major Revision.

We sincerely appreciate the opportunity to revise our manuscript. Following your suggestions, we have improved the literature review and made enhancements to the language.

In addition to the referees' suggestions, please:

1. Have a more comprehensive literature review to summarize the previous research and support the current research

We greatly appreciate this constructive feedback, which has made our article more comprehensive. In the **Introduction** section, we have added a literature review that elaborates on the definition, development, and clinical utility of PHLI. Additionally, we have summarized the current research directions of PHLI, the challenges encountered in clinical practice, and the existing research gaps that our study aims to fill, providing a basis for our research. (Please refer to pages 4-8, lines 47-131)

2. Seek professional editing services to enhance the readability of the work.

We appreciate your constructive suggestions and have sought the assistance of a professional editor to revise the language of the manuscript.

Reviewer #1: The term “chunk-check” can be more or less used in the software, programming and IT fields, but we are not aware of a specific meaning in the health field. The authors should therefore check its use and explain it or replace it with a more widely understood term.

Thank you for your kind reminder. Indeed, as you mentioned, the term "chunk-check" is more commonly used in the IT field, where it refers to breaking down large tasks into specific smaller chunks and checking each chunk sequentially. Now, this concept is also being applied to other fields. In the realm of health literacy, the application of "chunk-check" involves breaking down complex health information into smaller, more manageable parts and gradually checking for understanding to ensure the information is conveyed clearly and is easy to understand. [1] It helps to enhance the health decision-making capabilities of the audience.

Health literacy focuses on an individual's ability to obtain, understand, and use health information. Therefore, when dealing with health-related content, chunk-check method helps improve the effectiveness of information delivery. [2]

In this context, chunk-check typically involves the following aspects:

1. Chunking Information: Breaking down complex health information into smaller, more understandable segments. For example, dividing a complicated pharmaceutical explanation into shorter sentences or sections, allowing patients to absorb the information gradually.
2. Evaluating the Reasonableness of Chunks: Assessing whether these chunks are appropriate for the target audience, particularly the comprehension levels of psychiatric patients and their families. This includes checking sentence length, paragraph structure, word difficulty, and the logical flow and coherence of the information conveyed.
3. Assessing the Effectiveness of Information Delivery: Testing the patient’s understanding of the chunked health information through questioning and the teach-back method, and adjusting any content that is unclear or potentially misleading.

For instance, when writing instructions on titration therapy for depression patients, the chunk-check process involves dividing the instructions into different sections (such as definition, method, and efficacy observation) and using short sentences, clear steps, and concise language within each section. Then, we check if these sections are logical, appropriate for the reading levels of specific depression patients (e.g., those under 13 years old or over 65 years old), and if the educational objectives are met.

We sincerely appreciate your kind reminder, and we have added this explanation to the text. (Please refer to pages 5, lines 60-70)

“Pharmacists in pharmacy clinic create pharmaceutical files….”. We do not know whether this is a mistranslation to English, but it is not clear to us what is a “pharmacy clinic”. Similarly, we do not know what is a “public welfare free clinic”.

We apologize for the unclear expression. A more appropriate term than "pharmacy clinic" is "pharmacist-managed clinics" (PMC). [3] In China, PMC is not a standalone clinic but a specialized department within a hospital that provides outpatient pharmaceutical services. PMC is a healthcare method led by clinical pharmacists, aiming to offer services such as PHLI, medication therapy management, drug consultation, medication education, and medication safety guidance. The main responsibilities of PMC include: personalized medication plan development and optimization, monitoring and management of adverse drug reactions, polypharmacy management, chronic disease management, and patient medication education. This service model not only enhances patient medication safety and efficacy but also alleviates the burden on doctors and promotes collaborative healthcare. (Please refer to pages 11, lines 189-192)

"Public welfare free clinic" refers to free clinics organized by healthcare institutions, government bodies, or party organizations. In China, these free clinics are frequently held during holidays or significant commemorative days. They are not located in specific clinics but are set up in various locations such as plazas, hospital lobbies, community centers, and nursing homes. Renowned experts provide free medical consultations and treatments to local patients. Pharmacists also participate in these events, providing pharmaceutical health literacy interventions. We have already revised it in the manuscript to "free clinics." (Please refer to pages 15, lines 261-267)

Thank you for your reminder. We have improved the language expression and added explanatory notes.

We find very interesting the WeChat platform, it would be very useful if the authors could expand a bit its description for the uninformed readers.

We greatly appreciate your constructive suggestions, and we are pleased to see your interest in the WeChat platform. We have refined the discussion regarding it.

The WeChat is the largest mobile social platform in China and WeChat public account is an information subscription platform launched by it. Almost all medical institutions have established public accounts on WeChat. Due to the experience of internet-based appointment during the COVID-19 pandemic, patients in China have developed the habit of subscribing to hospital information through WeChat. Hospital WeChat public accounts mainly provide three types of content: online appointment and payment, dissemination of medical information, and publication of popular science articles. Patients can subscribe to articles about specific content related to the hospital on WeChat (The following image is a specific interface).

Research shows that the WeChat platform can be an effective intervention to alleviate anxiety in patients or caregivers, [4] and can also enhance the effectiveness of health literacy interventions, [5] and treatment outcomes. [6] Among these, popular science articles on WeChat play a significant role in patient health literacy interventions. Many healthcare professionals, including doctors, pharmacists, nurses, laboratory technicians, and counselors, publish popular science articles on WeChat public accounts, explaining diseases, medications, or treatments in simple language to improve public health literacy. Currently, articles on WeChat not only include text but also images (comics), videos, and games, which greatly promote pharmaceutical health literacy among the public (including patients). Short, independent popular science articles can be considered an important form of "chunk-check". As shown in the results section, the proportion of our hospital’s Internet information subscription mode in PHLI is steadily increasing, indicating that innovative PHLI methods such as WeChat popular science articles are playing an increasingly important role.

In those popular science articles, pharmacists break down complex pharmaceutical knowledge into chunks, using simple language and images to simplify difficult-to-understand information. For example, when explaining the relationship between pharmacogenomics and depression treatment, pharmacists often start with a case that highlights varying responses to the same antidepressant among different patients, pointing out the potential role of pharmacogenomics. Next, pharmacists use images or videos to explain medical terms such as pharmacogenomics, genotype, and cytochrome P450 enzyme. They then describe the definition of metabolism and how different genotypes affect it. Finally, pharmacists emphasize how genetic testing determines a patient's genotype to guide medication use. These articles are published as a series, and comments below the articles are collected. Pharmacists adjust the plans of the articles based on those comments to address information gaps in a timely and accurate manner, completing the self-iterative function of the internet information subscription model.

Similar to your feelings, we also found that WeChat platform plays a unique role in PHLI. Thank you again for this constructive suggestion. (Please refer to pages 12-13, lines 212-227)

“The third was intervention based on instant messaging software. Many elderly people in China found it challenging to conduct online consultations with pharmacies. As a result, pharmacists can communicate via instant messaging software with patients, allowing them to monitor the patients’ medications and provide real-time dynamic PHLI as needed.” This is actually very vague: what instant messaging software was used? How where the patients having access to the pharmacists for messaging? How frequent were the interactions?

We apologize for any unclear statements and have refined this section to better address your concerns. Instant messaging software in our context primarily refers to WeChat. In China, WeChat functions both as an institutional platform for publishing and subscribing to information and as an instant messaging tool for personal communication. While some older adults may struggle with reading WeChat public account articles or navigating complex online medical interfaces, they primarily use WeChat for personal communication. We find that using WeChat allows us to reach this demographic, overcoming spatial limitations and delivering high-quality PHLI to improve outcomes. Through WeChat, patients can establish one-on-one contact with pharmacists. If necessary, pharmacists can access patients' medication information and provide monitoring. Therefore, if the PHLI is initiated passively by the patient, it often does not follow a fixed frequency and pharmacists providing immediate PHLI services as needed. However, if the pharmacist actively initiates the monitoring service for patients, interactions are conducted daily. (Please refer to pages 13-14, lines 237-244)

In the Results section, the authors only provide numbers of patients on whom one intervention was carried out. However, it would be useful to know what was the total number of the pool of patients out of whom those interventions were applied (i.e. how frequent was a PHLI intervention among inpatients? how frequent was among outpatients? Etc).

We appreciate your professional suggestion and have added the total number of the pool of patients in parentheses after each case in Table 1. The details are as follows: (Please refer to pages 17, Table 1)

Table 1 The number of PHLI cases in different ways

| Types | 2022 |  | 2023 |  | Total |  |
| --- | --- | --- | --- | --- | --- | --- |
|  | Cases | Ratio/% | Cases | Ratio/% | Cases | Ratio/% |
| PHLI mode based on inpatient | 165 (327) | 52.38 | 221 (378) | 68.85 | 386 (705) | 60.69 |
| PHLI mode based on pharmacist-managed clinics | 57 (63) | 18.10 | 8 (10) | 2.49 | 65 (73) | 10.22 |
| PHLI mode based on TDM and gene detection | 28 (30) | 8.89 | 14 (25) | 4.36 | 42 (55) | 6.60 |
| PHLI mode based on Internet information subscription | 35 (38) | 11.11 | 56 (59) | 17.45 | 91 (97) | 14.31 |
| PHLI mode based on Internet hospitals | 18 (22) | 5.71 | 10 (13) | 3.12 | 28 (35) | 4.40 |
| PHLI mode based on instant messaging software | 3 (5) | 0.95 | 4 (9) | 1.25 | 7 (14) | 1.10 |
| PHLI mode based on Community | 9 (19) | 2.86 | 8 (33) | 2.49 | 17 (52) | 2.67 |
| Total | 315 (504) | 100.00 | 321 (527) | 100.00 | 636 (1031) | 100.00 |

“Among them, 200 cases ranged from 18 to 40 years old, accounting for 37.88 % (Table 2).” “Among them” refers to patients from 2022, from 2023 or the total number of the two years?

We apologize for the unclear expression. The 200 cases refer to the total number of 2022 and 2023, specifically the sum of the numbers highlighted in red in the following table (Table 2). The details are as follows: (Please refer to pages 17, lines 308-309)

Table 2 Basic information of the patients

|  | Gender | Age |  |  |  |  |  |  |  | Total | Ratio/% |
| --- | --- | --- | --- | --- | --- | --- | --- | --- | --- | --- | --- |
|  |  | ≤17 | 18-30 | 31-40 | 41-50 | 51-60 | 61-70 | 71-80 | ≥81 |  |  |
| 2022 | Male | 11 | 27 | 20 | 20 | 16 | 15 | 5 | 0 | 114 | 42.07 |
|  | Female | 20 | 47 | 25 | 10 | 26 | 22 | 3 | 4 | 157 | 57.93 |
| 2023 | Male | 9 | 16 | 19 | 12 | 16 | 10 | 13 | 5 | 100 | 38.91 |
|  | Female | 37 | 26 | 20 | 19 | 13 | 23 | 10 | 9 | 157 | 61.09 |
| Total |  | 77 | 116 | 84 | 61 | 71 | 70 | 31 | 18 | 528 |  |
| Ratio/% |  | 14.58 | 21.97 | 15.91 | 11.55 | 13.45 | 13.26 | 5.87 | 3.41 | 100.00 |  |

“Among them, pharmaceutical care accounted for 7.17 % of PHLI in 2023, with 23 cases.” How was pharmaceutical care defined in this context?

Pharmaceutical care refers to the patient-centered pharmaceutical services provided by clinical pharmacists. In China, these services include a comprehensive range of medication-related plans, such as medication reconciliation, drug therapy assessment, therapeutic drug monitoring (TDM), medication education and guidance, and adverse drug reaction management. These services are often tailored for patients on multiple medications, older adults, children, those with chronic diseases, special physiological conditions, severe comorbidities, or a history of adverse drug reactions.

When it is determined that a patient needs medication monitoring, pharmacists simultaneously initiate PHLI. They communicate information about pharmaceutical care through various means (such as written, verbal, and visual) to help patients make informed decisions about their medication therapy and health. It is evident that in 2023, the PHLI in the field of pharmaceutical care at our hospital has increased significantly compared to 2022. We have made the corresponding revisions in the text. (Please refer to pages 18, lines 314-315)

A number of the Types listed in Table 3 need clarification for the interested readers: what does “Usage of special dosage forms” refer to? “Medical science popularization“? “Drug gene”? (what was the pharmacist activity relating to the “drug genes”?) “Pharmaceutical care”? “Index interpretation“? “Prescription issued“?

The same holds true for Types listed in Table 4: “Interpretation of Pharmaceutical Report“? “Regular inspections”? “Avoid grinding to take”? “Drug difference”? “Avoid random reduction“? “Avoid random additions”? “Active movement“? “The difference of treatment plan”?

We apologize for the poor language. Table 3 refers to the types of PHLI, specifically the distribution of fields where pharmacists engage in PHLI. This includes interventions related to adverse reaction, dosage and administration, TDM, and other fields. Table 4, on the other hand, refers to the distribution of different intervention strategies. This includes adverse reaction identification, interpretation of pharmaceutical report, routine examination reminder, and more. It is important to note that different fields of PHLI may involve the same intervention strategies. For instance, strategies for addressing patient concerns and adverse reactions might both include identifying adverse reactions. Similarly, the same field can have different intervention strategies, such as adverse reaction interventions, which may include avoiding sudden discontinuation of medication and providing regular check-up reminders.

The explanations of some terms in Table 3 are as follows:

1. **Special dosage form administration** (previously "**Usage of special dosage forms**") refers to the PHLI provided by pharmacists to patients regarding the use of special dosage forms, such as controlled-release formulations or orally disintegrating tablets. Intervention strategies include avoiding the crushing of medication, changing the type of medication, etc. (as detailed in Table 4).
2. **Medical science popularization** refers to pharmacists writing popular science articles as part of PHLI. The strategies corresponding to the content of these articles include promoting physical activity, avoiding missed doses, and more (as detailed in Table 4).
3. **Pharmacogenomics** (previously "**Drug gene**") refers to PHLI conducted by pharmacists focusing on genetic information. Intervention strategies include addressing drug differences based on the patient’s genotype report (as detailed in Table 4). The role of pharmacists in pharmacogenomics includes interpreting genetic reports, providing professional information to physicians (such as metabolizer status and drug selection strategies), and conducting PHLI related to pharmacogenomics for patients (including the significance of genetic testing and explanations of drug-metabolizing enzymes).
4. **Pharmaceutical care** refers to the PHLI conducted by clinical pharmacists before providing patient-centered pharmaceutical services.
5. **Test result interpretation** (previously "**Index interpretation**") refers to the PHLI conducted by pharmacists regarding the medical terminology and test result, such as interpreting bacterial culture reports.
6. **Prescription issuance rules** (previously "**Prescription issued**") refers to the PHLI conducted by pharmacists regarding the rules for issuing prescriptions. This includes health literacy interventions related to prescription rules for medications such as benzodiazepine and psychotropic drugs in China. Currently, China has strict prescription management for such drugs (which are addictive and prone to dependence), allowing only a limited quantity to be prescribed for specific indications. Consequently, patients often seek advice on these regulations.

The explanations of some terms in Table 4 are as follows:

1. **Interpretation of pharmaceutical report** refers to the pharmacist's interpretation of patients' pharmaceutical reports, such as explaining blood drug concentration reports within normal ranges that require no intervention and are provided upon request.
2. **Routine examination reminders** (previously "**Regular inspection**") refers to the intervention strategy where pharmacists remind patients to undergo regular checks for certain indicators. This often applies to the following groups of patients: those who have experienced adverse reactions, children taking medications, and patients with drug interactions.
3. **Avoidance of crushing medication** (previously "**Avoid grinding to take**") refers to the intervention strategy for patients taking medications such as extended-release tablets, advising them to avoid crushing these medications.
4. **Popular science on drug distinctions** (previously "**Drug difference**") refers to informing patients about the differences between medications, including explaining the distinctions between brand-name drugs and generic drugs.
5. **Avoidance of arbitrary dose reduction** (previously "**Avoid random reduction**") refers to the intervention strategy for patients who are experiencing inadequate medication efficacy but have not yet reached an effective dose or have changed their medication brand. The strategy involves advising patients to avoid arbitrary dose reductions.
6. **Avoidance of arbitrary dose escalation** (previously "**Avoid random additions**") refers to the intervention strategy for patients who may have a tendency for misuse or those who have switched to generic drugs. The strategy involves advising patients to avoid arbitrary dose increases.
7. **Regular exercise** (previously "**Active movement**") refers to the intervention strategy of reminding patients, for whom exercise therapy is a first-line treatment, to engage in regular physical activity.
8. **Popular science on treatment plan distinctions** (previously "**The difference of treatment plan**") refers to the intervention strategy for patients who are confused about different treatment plans. This involves explaining the distinctions between various treatment options.

To address your concerns, we have revised the language in the tables. (Please refer to pages 18-20, Table 4, Table 5)

It would have been useful to also know how many pharmacists were involved in those interventions. Was there any additional training before starting the interventions? Was there any guidance/documentation available for pharmacists to ensure consistent and uniform provision of the interventions? What would be the average time for an intervention? (possibly by types of interventions).

We appreciate your professional suggestions and have improved the content of the article accordingly. Five clinical pharmacists were involved in this model. Before starting the interventions, all pharmacists received clinical pharmacist training and certification from the National Health Commission. Additionally, the pharmacists underwent extensive training in communication with patients. We developed the " Standard Operating Procedure for Pharmaceutical Management and Pharmaceutical Services at The Affiliated Mental Health Center of Jiangnan University" which includes items on pharmaceutical health literacy such as communication strategies, encouraging patient questions, and techniques for PHLI. Prior to implementing the intervention model, we also conducted targeted training on PHLI, covering basic methods such as teach-back, chunk-check, and simple language to ensure the standardization and accuracy of pharmacist interventions.

Indeed, as you mentioned, the duration of interventions is uncertain and depends on various factors. For patients requiring pharmaceutical monitoring, the time for PHLI is relatively longer, whereas for PHLI on different dosage forms (e.g., the use of orally disintegrating tablets), the time is often shorter. PHLI interventions based on Internet hospital can only be conducted when pharmacists are logged into the online medical system; interventions based on WeChat tend to be shorter. Additionally, publishing popular science articles takes more time, while PHLI conducted in pharmacist-managed clinics is relatively quicker since face-to-face verbal interventions can convey more content.

In fact, at the outset of implementing PHLI, we considered including intervention duration in our study. However, based on preliminary statistics, we found that the duration of interventions depends not only on the different types of interventions mentioned but also on factors such as the patient's level of pharmaceutical health literacy, demographic characteristics like age and educational background, the patient's curiosity, and whether the patient initiates the PHLI. Therefore, at this stage, our research focuses on intervention strategies and types. We aim to quickly promote this model and provide the most accurate insights and valuable perspectives for medical institutions and healthcare professionals that have not yet implemented PHLI. As a result, we have not yet conducted statistics on the duration of PHLI.

Your suggestions have been very insightful and inspiring for us. Moving forward, we plan to implement two initiatives. First, we will include a broader population in our PHLI work, assessing patient satisfaction, evaluating the effectiveness of interventions, and tracking intervention duration. We will conduct a comprehensive analysis considering patient demographics and intervention types, as we believe that demographic-based analysis will provide more meaningful insights. Second, based on the overall population, we will expand to include specific subgroups, such as patients with late-life depression, to refine PHLI intervention strategies. This will help enrich the PHLI model database and lay the foundation for future precise predictions. The first research initiative has already been submitted to the ethics committee of our hospital and will proceed once approved. (Please refer to pages 8-9, lines 139-147)

Moderate changes of English necessary (agreements between nouns and verbs etc).

We apologize for the poor English. Thank you for your professional advice; we have thoroughly revised the English text.

Reviewer #2: I think authors need to include more information in the introduction and explain more about statistical analysis they used. English language was good.

Thank you for your constructive suggestions. We have added a literature review in the **Introduction** section, summarizing the definition, development, clinical applications, current research focus, and gaps related to PHLI. We have reviewed previous research experiences and identified gaps in the field to support our study. Additionally, we have refined the analysis of statistical methods in the **Methods** section. (Please refer to pages 4-8, lines 47-131, pages 15-16, lines 274-280)

Reference

1.	**The Health Literacy Place. Chunk and check. Available at:** [<https://www.healthliteracyplace.org.uk/toolkit/techniques/chunk-and-check/>]

2.	Cork T, White S: **Exploring community pharmacists' use of health literacy interventions in their everyday practice**. *Research in social & administrative pharmacy : RSAP* 2022, **18**(11):3948-3952.

3.	Cai M, Zhou L, Gao D, Mei D, Zhang B, Zuo W, Yan Q: **A national survey of individualized pharmaceutical care practice in Chinese hospitals in 2019**. *Frontiers in pharmacology* 2023, **14**:1022134.

4.	Wu J, Meng J, Li H: **WeChat-platform-based education and care program as a candidate approach to relieve anxiety, depression, and post-traumatic stress disorder in parents of pediatric and adolescent patients with osteosarcoma**. *Frontiers in psychology* 2022, **13**:913940.

5.	Petkovic J, Duench S, Trawin J, Dewidar O, Pardo Pardo J, Simeon R, DesMeules M, Gagnon D, Hatcher Roberts J, Hossain A *et al*: **Behavioural interventions delivered through interactive social media for health behaviour change, health outcomes, and health equity in the adult population**. *The Cochrane database of systematic reviews* 2021, **5**(5):Cd012932.

6.	Bi J, Yang W, Hao P, Zhao Y, Wei D, Sun Y, Lin Y, Sun M, Chen X, Luo X *et al*: **WeChat as a Platform for Baduanjin Intervention in Patients With Stable Chronic Obstructive Pulmonary Disease in China: Retrospective Randomized Controlled Trial**. *JMIR mHealth and uHealth* 2021, **9**(2):e23548.
